# Experimental Study of the Airflow Field and Fiber Motion in the Melt-Blowing Process

**DOI:** 10.3390/polym16040469

**Published:** 2024-02-07

**Authors:** Wenhan Wu, Wanli Han, Yafeng Sun, Honglei Yi, Xinhou Wang

**Affiliations:** 1 College of Material and Textile, Jiaxing University, Jiaxing 314000, China; 00188126@zjxu.edu.cn (W.W.); yi-hl@163.com (H.Y.); 2 Jiaxing COETEKS Material Co., Ltd., Jiaxing 314000, China; yafeng.sun@coeteks.cn; 3 College of Mechanical Engineering, Donghua University, Shanghai 201620, China; xhwang@dhu.edu.cn

**Keywords:** melt blowing, airflow field distribution, fiber motion, experimental measurement

## Abstract

The melt-blowing process involves high velocity airflow and fiber motion, which have a significant effect on fiber attenuation. In this paper, the three-dimensional airflow field for a melt-blowing slot die was measured using the hot-wire anemometry in an experiment. The fiber motion was captured online using a high-speed camera. The characteristics of the airflow distribution and fiber motion were analyzed. The results show that the melt-blowing airflow field is asymmetrically distributed. The centerline air velocity is higher than that around it and decays quickly. The maximum airflow velocity exists near the die face, in the range of 130–160 m/s. In the region of −0.3 cm < *y* < 0.3 cm and 0 < *z* < 2 cm, the airflow has a high velocity (>100 m/s). As the distance of *z* reaches 5 cm and 7 cm, the maximum airflow velocity reduces to 70 m/s. The amplitude of fibers is calculated, and it increases with the increase in air dispersion area which has a significant influence on fiber attenuation. At *z* = 1.5 cm, 2.5 cm, 4 cm, and 5.5 cm, the average fiber amplitudes are 1.05 mm, 1.71 mm, 2.83 mm, and 3.97 mm, respectively. In the vicinity of the die, the fibers move vertically downward as straight segments. With the increase in distance from the spinneret, the fiber appears to bend significantly and forms a fiber loop. The fiber loop morphology affects the velocity of the fiber movement, causing crossover, folding, and bonding of the moving fiber. The study investigated the interaction between the fiber and airflow fields. It indicates that the airflow velocity, velocity difference, and dispersion area can affect the motion of fiber which plays an important role in fiber attenuation during the melt-blowing process.

## 1. Introduction

Nonwovens are commonly defined as manufactured sheets or webs of randomly oriented fibers, which are entangled or adhered by fibers without requiring any downstream processing such as weaving or knitting. One of the most significant developments of nonwoven technology is melt blowing, which involves applying a hot airflow to an extruding polymer melt and drawing the polymer jet into microfibers. Melt blowing is a single-step process, a high-throughput and economical approach widely used in the production of synthetic nonwovens. Accordingly, this process accounts for more than 10% of global production of nonwovens [1,2,3,4]. At present, the fineness of melt-blowing fibers is generally 1–5 um, which has been widely used in protective materials, filtration materials, oil-absorbing materials, thermal materials, battery separators, and other fields [5,6,7,8,9,10,11,12].

In the melt-blowing process, the high-velocity, hot-air jet rapidly attenuates the molten stream of polymer into fibers. The melt-blowing technology is rather a complex process, in which there are many different variables having impact on fiber formation such as polymer properties, polymer distribution die, air velocity, air pressure, air temperature, die-to-collector distance, die temperature, and type of dies [13,14,15]. Over the past decades, a considerable amount of theoretical and experimental research has been conducted on melt-blowing technology. Most research is focused on the melt-blowing coat-hanger die distribution [16,17], the air drawing of the polymer [18,19], the performance of the melt-blowing dies [20,21], and the fiber movement [22,23].

It is known that the airflow field plays an important role on fiber formation in the melt-blowing process. Knowledge of the velocity distribution of the airflow field is of vital importance for predicting the performance of the die, understanding the melt-blowing process, and modeling of the fiber formation process [15]. Many researchers have conducted a lot of work on the airflow field with computational fluid dynamics (CFD) and experimental investigation. CFD is widely used to investigate the melt-blowing airflow field and to optimize the melt-blowing die, owing to its advantage of cost saving and convenience. Shambaugh and coworkers [24,25,26] have finished a series of work on understanding the airflow field in the melt-blowing process with techniques in computational fluid dynamics. Chelikani [27] used a numerical simulation method to demonstrate that the Coanda effect would influence the trajectory of fine fibers created by the melt-blowing process. Wang investigated the common slot die parameters such as slot width, nose-piece width, slot angle, and setback on the total airflow field. They utilized the CFD approach and optimization methods to obtain the theoretically optimum configuration of the common slot die [28,29]. The CFD approach can also provide other important parameters that are not easily measured experimentally, such as turbulent intensity, dissipation rate, and air-flow field profiles for multiple die configurations in order to identify favorable design parameters. However, there are some differences between the numerical simulation method of the melt-blown airflow field and the actual melt-blown airflow field. This is because the conditions of the airflow field in the numerical simulation are idealized, while the experimental conditions in practice are often subject to changes in external conditions. For the experimental work, it mainly focuses on the measurement of the mean velocity field and the mean temperature field in the melt-blowing process. Shambaugh [30,31,32] also have carried out a series of experiments to measure the airflow field below a melt-blowing slot die, using a pitot tube and a thermocouple or infrared thermometer. The regression equation of the airflow velocity distribution is calculated using a statistical analysis approach in their research. Zeng [33,34] have introduced the hot-wire anemometer (HWA) with a single hot-wire and two-wire HWA to measure the turbulent airflow field and analyzed the fiber motion in the melt-blowing process. Xie [35] measured the turbulent airflow in melt-blowing slot die online with the approach of the Particle Image Velocimetry (PIV) technique. The measured data can verify the numerical simulation results and also analyze the fiber drawing process. The information related to the application of the experimental method to measure the melt-blowing airflow field is shown in Table 1.

From Table 1, it is clear that the experimental measurements of the melt-blowing airflow field have been relatively mature, and the characteristics of the flow field are basically clear. The hot-wire anemometer is widely used in the melt-blowing airflow field measurement experiments. This equipment is highly sensitive and can measure very small velocity pulsations with high measurement accuracy and good repeatability. However, most experimental measurements of the melt-blown airflow field have only measured the average values of the velocity and temperature of the airflow field, while the velocity distribution characteristics of the airflow field have not been measured and analyzed. In addition, there are relatively few studies on the fiber motion in the melt-blowing process, such as the fiber motion state in the airflow field and the effect of fiber motion on the melt-blowing web structure have not been reported.

In this paper, the three-dimensional velocity field of the melt-blowing airflow was measured using the hot-wire anemometer in an experiment. The fiber motion in the airflow field was captured using a high-speed camera and analyzed. This study investigated the interaction between the fiber and the airflow fields. Moreover, the velocity distribution in the airflow field and the velocities at different positions were analyzed. The motion state and the drawing process of fiber in the airflow field are explained. The effect of the change in fiber motion in the airflow field on the fiber arrangement form in the fiber web is also explained. Experimental methods to fully understand the melt-blowing airflow field and fiber movement will help to enrich the melt-blowing technology.

## 2. Experiment and Materials

### 2.1. Experimental Setup

**Figure 1:**
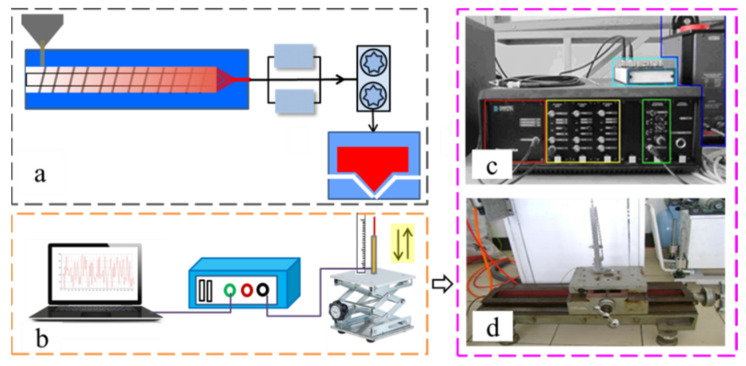
Schematic diagram of the experimental setup for the melt-blowing airflow field. (**a**) Melt-blowing equipment. (**b**) The airflow field measuring system. (**c**) The hot-wire anemometer device. (**d**) The traverse mechanism equipment.

Figure 1 depicts the experimental setup for the three-dimensional airflow field measurement in the melt-blowing process. It consisted of melt-blown equipment and a measuring system. The melt-blowing die is a slot die which can produce two high-speed air jets to stretch the melt polymer jet into ultrafine fibers, as shown in Figure 1a. The measuring system has the hot-wire anemometer which is produced by Dantec Company, Copenhagen, The Kingdom of Denmark, model Dantec CTA/HWA (Streamline) and the traverse mechanism equipment in Figure 1b. The hot-wire anemometer includes a hot-wire host (Dantec StreamLine Frame 90N10), velocity measurement module (Dantec StreamLine CTA90C10), streamline CTA 90C20), and calibrator (Dantec Calibration System 90H10) in Figure 1c. The installation and disassembly between modules are convenient and easy to operate. Figure 1d is the traverse mechanism equipment. It can fix the coordinate frame which is employed to ensure the displacement of the hot-wire probe during the measuring procedure. The traverse mechanism equipment can move in three dimensions with an accuracy of 0.01 mm. The directional movement is realized by the rotation of the screw.

### 2.2. Melt-Blowing Die and Airflow Measure Parameter

**Figure 2:**
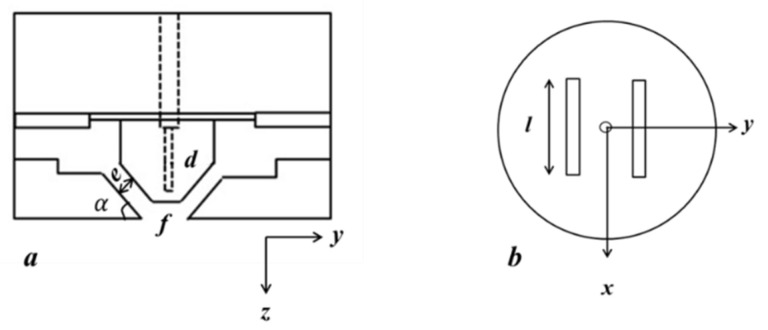
Schematic of the melt-blowing die (**a**) cross-section view and (**b**) end-on view.

In the melt-blowing process, a stream of hot polymer is extruded from the die spinneret and enters into the high-velocity and hot-temperature airflow field. The force of the air jets upon the polymer results in the rapid attenuation of the polymer into fine fiber. The velocity of airflow gradually decreases with the distance of the collector increasing. In this experiment, a single-orifice, melt-blowing device was used to measure the airflow field and observe the fiber motion of melt-blowing process. The schematics of slot die, and the die dimensions are shown in Figure 2 and listed in Table 2.

**Figure 3:**
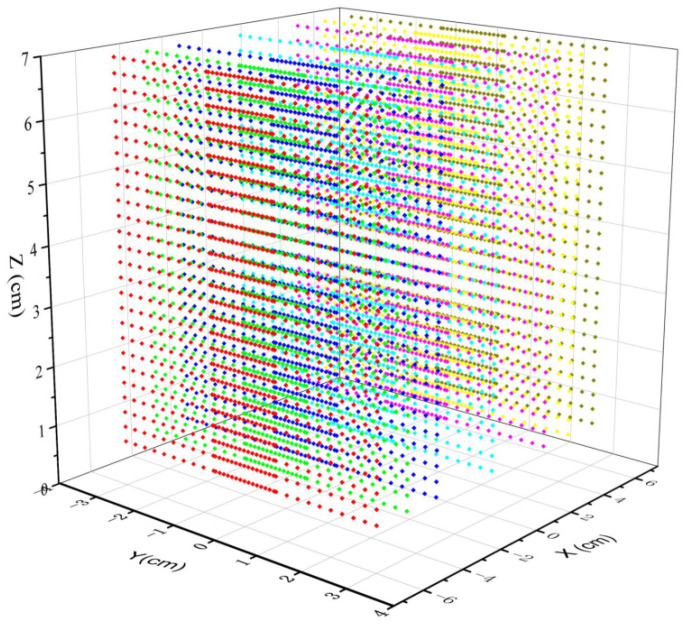
The coordinate system and measuring point of airflow field measurement.

The centerline airflow field plays a significant role because polymer streams spend most of the time in the airflow field. In order to better obtain the distribution characteristics of the airflow field, the measurement points for the centerline airflow field should be the key measure area, while the areas away from the spinning line can have fewer measurement points. The measurement area for this experiment was 6.25 cm × 6 cm × 12 cm, 0.75 cm ≤ *z* ≤ 7 cm, −3 cm≤ *y* ≤ 3 cm, −6 cm ≤ *x* ≤ 6 cm. The starting point of the measurement in the *z*-axis direction cannot be zero. It is due to being too close to the die spinneret. The airflow velocity is too large, causing the hot wire to break. The vertical direction *z* displacement interval is 2.5 mm, a total of 26 data points. In the y-axis direction, the measurement spacing in the interval [−0.7, 0.7] is 0.1 mm, and the total number of measurement points is 35. The measurement spacing in the rest of the y-axis interval is 2.5 mm. The *x*-axis direction measurement points are 7 sets of data, in the range of [−6, 6] interval of 1.5 mm. The distribution of the measured data point is shown in Figure 3. The different colored dots represent measurement data at different surfaces locations.

### 2.3. Hot-Wire Anemometer Calibrations

The performance of the hot-wire probe is affected by the manufacturing process and metal material, as well external conditions such as fluid temperature, density, and velocity. In experiments, the hot-wire probe should be calibrated in order to obtain accurate measurements [34,41]. For velocity calibration, the calibration device provided air to blow the wire with different velocities. The corresponding voltages applied to the wire have been collected to maintain its temperature. The rated velocities and voltages are fitted into quartic polynomial equation and it is called the velocity calibration curve. In this experiment, Dantec Calibration System 90H10 was used for the Dantec hot-wire anemometer. The designed velocities were selected to calibrate the experiment with the 55P11 probe. The calibrated maximum speed is 200 m/s, and the minimum speed is 30 m/s.

### 2.4. Process Parameter and Material

In this experiment, the isotactic polypropylene (PP) polymer was used, and the melt flow rate was 350 g/10 min. The PP is fed to the extruder through the hopper. The motor-driven screw pushes the material through the extruder and the polymer flow rate is 6.5 mL/min. The screw had four heating zones and the temperatures of the four heating zones were 200, 220, 240, and 260 °C, respectively. The temperatures of both the die and the heated air were 260 °C. The melt-blown fibers were collected by a stainless steel screen with a speed of 10 m/min. The collector distance was 25 cm. In the experiment, the fiber trajectory was captured by the Redlake camera during the operation of the melt-blowing equipment. The experimental high-speed camera had a frame rate of 5000 frames/s, an exposure time of 0.2 ms, and a recording time of 0.2 s. The fiber morphology were observed using scanning electron microscopy (SEM; Hitachi S-4800, Tokyo, Japan) at 12 keV after coating with a gold layer.

## 3. Results and Discussion

### 3.1. The Melt-Blowing Airflow Field Distribution

**Figure 4:**
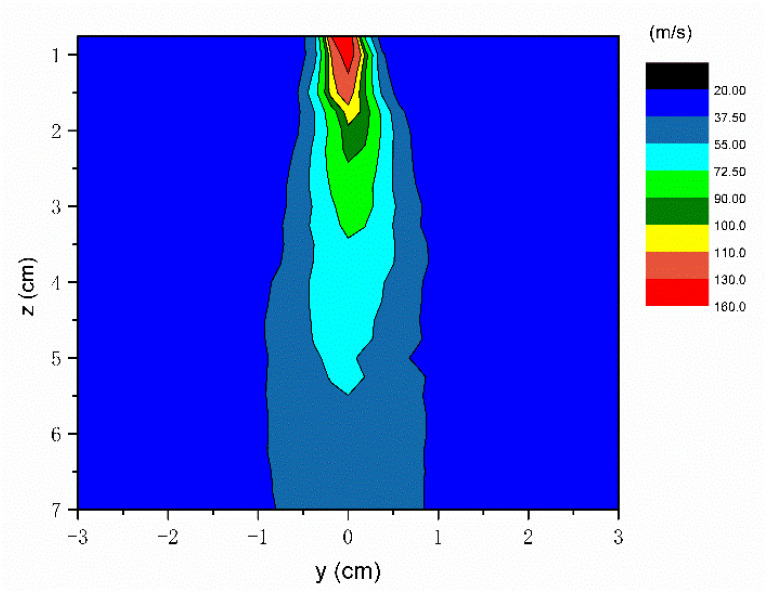
The airflow field on the *yz* plane for the melt-blowing process for *x* = 0.

Figure 4 shows the velocity contour maps of *yz* plane airflow field at *x* = 0. It indicates that the experimentally measured airflow field differs from the numerically simulated airflow field. The velocity distribution is not symmetrically distributed. The airflow has fluctuated in the direction of the centerline plane. The numerical simulation of the melt-blowing airflow field distribution is the result obtained under ideal conditions. The boundary conditions, initial conditions, and iterative results of the simulation process are fixed and are not disturbed by the external environment. In contrast, the high-velocity airflow from the die slot will cause fluctuations in the surrounding airflow below the melt-blowing die in the experiment. It will interfere with the air jet and affect its symmetrical distribution. In the region of −0.3 cm < *y* < 0.3 cm and 0 < *z* < 2 cm, the airflow has a high velocity (>100 m/s). The maximum airflow velocity exists near the die face, in the range of 130–160 m/s, with the red air velocity contour map. In this region, the two air jets interact with each other and can obtain the combined point where the mean streamwise velocity gets its maximum value on the instant. The airflow close to the die is very important to the fiber formation in the melt-blowing process. The fibers stay in the center of the airflow field most of time. The main drag force for the fiber attenuation comes from the high-velocity air jets. The reason is that there is a velocity difference between the airflow and the fiber. In the region of −0.5 cm < *y* < 0.5 cm and 0 < *z* < 3.5 cm, the air velocity is above 75 m/s. The high air velocity is favorable for fiber attenuation in this region. As shown in Figure 4, the velocity of the airflow decreases from 150 m/s to 70 m/s at *z* = 5.4 cm. It shows that the airflow velocity decays rapidly to dissipate after *z* > 5 cm.

**Figure 5:**
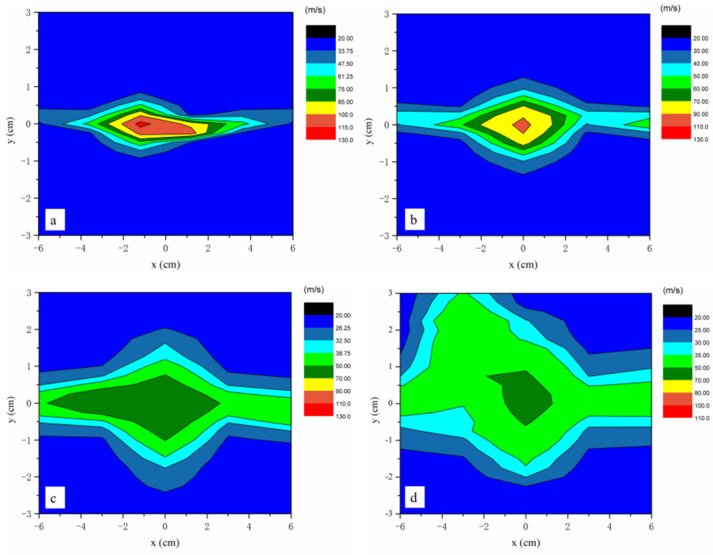
The airflow field at various positions along the z-axis is depicted on the *xy* plane. (**a**) The airflow field distribution at the distance of *z* = 1.75 cm. (**b**) The airflow field distribution at the distance of *z* = 2.75 cm. (**c**) The airflow field distribution at the distance of *z* = 5 cm. (**d**) The airflow field distribution at the distance of *z* = 7 cm.

Figure 5 shows the xy plane velocity contour at different locations along the z-axis. In this experiment, the direction of the die slot is parallel to the x-axis and perpendicular to the *y*-axis. The distribution of airflow is narrower in the *y*-axis and wider in the *x*-axis. Figure 5 also shows that all airflow fields at the different *z* distances are distributed irregularly due to the fluctuations in the airflow field.

Figure 5a shows the air velocity contour maps of *xy* plane at the distance *z* = 1.75 cm. The air is ejected from the slots of the melt-blowing die, and it does not disperse, maintaining a high velocity for a short distance. In the region of −2 cm < *x* < 2 cm and −0.5 cm < *y* < 0.5 cm, the velocity is above 100 m/s with a small distribution area. When the distance is *z* = 2.75 cm, the velocity distribution area of 100 m/s becomes smaller and the velocity distribution area of 40–90 m/s increases significantly, as shown in Figure 5b. The airflow disperses in the surrounding area with the z-axis increasing. As the distance of *z* reaches 5 cm and 7 cm, the maximum airflow velocity reduces to 70 m/s in Figure 5c,d. The velocity distribution area also becomes larger, as shown in Figure 5c,d. Near the melt-blowing die, the airflow velocity is larger compared to the fiber velocity. There is a large velocity difference between the airflow and the fiber, which is beneficial to fiber attenuation. The results of the experiment show that the airflow decay rate becomes smaller and spreads to a larger airflow distribution area with the *z*-axis distance increasing, which causes fiber fluctuations in the melt-blowing process.

### 3.2. The Air Velocity Distribution in the Melt-Blowing Process

**Figure 6:**
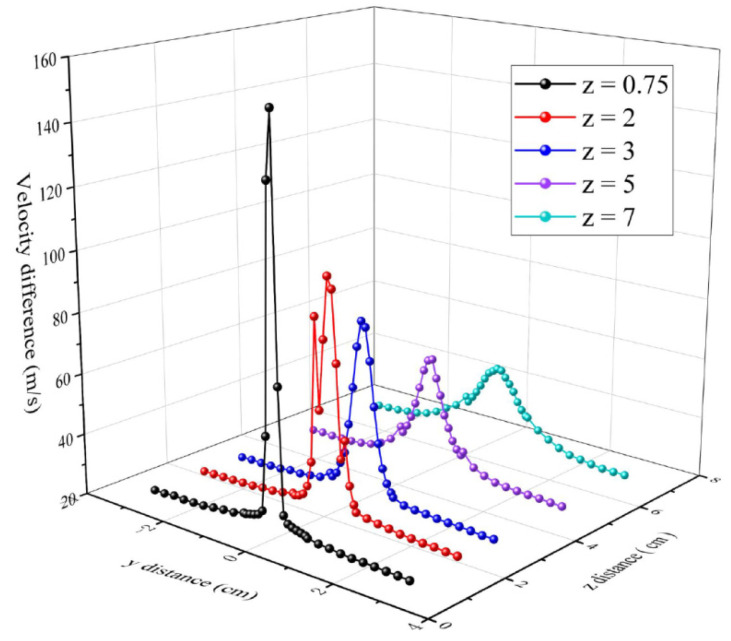
The distributions of air velocity at different *z*-axis position along the *y*-axis.

Figure 6 is the air velocity on the central surface of the melt-blowing airflow field (*y*-*z* plane at *x* = 0) at different *z* positions. At the beginning, the peak of the airflow is the largest and the velocity distribution is narrow. The distribution of airflow is mainly concentrated on the centerline. The distribution of the airflow becomes wider along the *y*-axis with the *z*-axis distance increasing. The variations of the air velocity along the y-direction are mainly distributed in the region −1 cm < *y* < 1 cm. When the *y* distance exceeds 2 cm from the center point, the measured velocity remains stable at about 21 m/s. The high velocity distribution of the airflow is mainly concentrated in the [−2, 2] *y*-axis region. Moreover, Figure 6 shows that the velocity of the flow field decays rapidly in the *z* direction. At *z* = 0.75 cm, the experimentally measured maximum velocity is 151.5 m/s, while the maximum velocity is 36.1 m/s at *z* = 7 cm. It can be seen that the airflow velocity decays rapidly over a relatively short distance. The magnitude of the air velocity is an important factor affecting fiber attenuation. Shambaugh has stated that the melt-blown fiber attenuation distance was in the range of 5 cm, and the experimental data in this experiment are consistent to his study.

**Figure 7:**
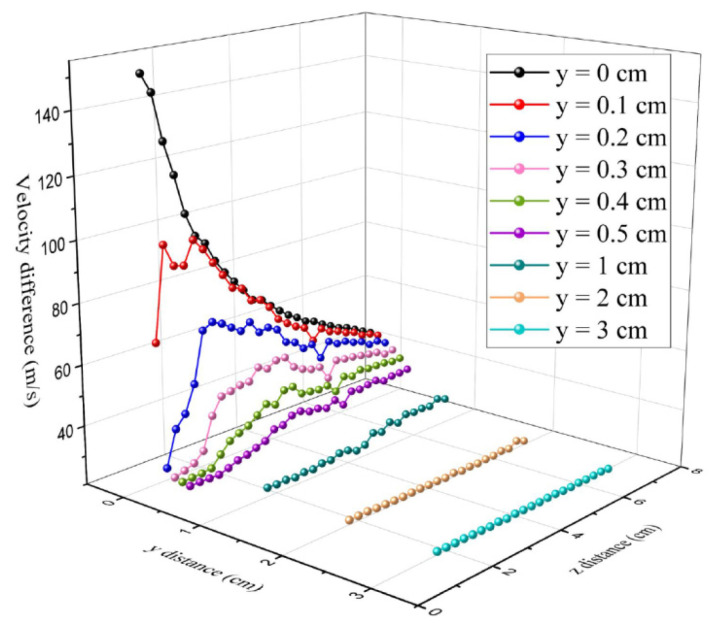
The distributions of air velocity at the different *y*-axis positions along *z*-axis.

The distributions in air velocity of the *y*-axis along *z*-axis are shown in Figure 7. When *y* = 0 cm, the centerline airflow velocity is the maximum at the initial point *z* = 0.75 cm. The airflow velocity decreases as the *z*-axis increases. At 0.1 cm < *y* < 0.5 cm distance, the *y*-axis airflow velocity is first increasing and then decreasing as the *z*-axis increases. At *y* = 0.1 cm, 0.2 cm, 0.3 cm, 0.4 cm, and 0.5 cm, the maximum velocity of the airflow is 151.50 m/s, 98.71 m/s, 70.85 m/s, 52.59 m/s, 43.20 m/s, 36.21 m/s at *z* = 0.75 cm, 1 cm, 2 cm, 3.5 cm, 3.75 cm, and 6 cm, respectively. The maximum airflow velocity for the *y*-axis position is different and the greater velocity on the *y*-axis closes to the centerline position. It can be seen that the high-velocity airflow is mainly concentrated in a narrow area just below the spinneret and attenuates quickly. The variations and fluctuations of velocity in airflow field can affect fiber drawing and motion trajectories.

**Figure 8:**
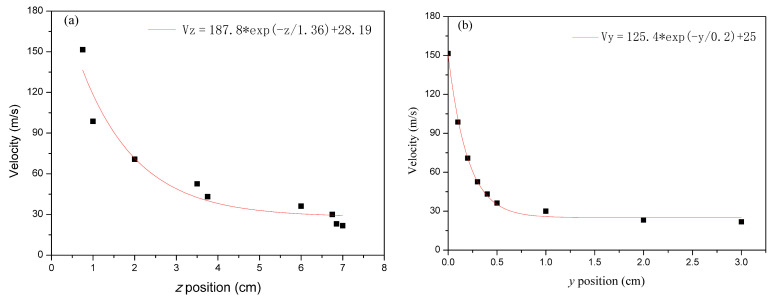
The maximum velocity distribution along the *z*-axis (**a**) and the *y*-axis (**b**) in the airflow field.

Figure 8 shows the maximum air-velocity distribution at different axis positions. Figure 8a shows the air velocity along the *z*-axis and Figure 8b shows the air velocity along the *y*-axis. Figure 8 shows an exponential trend decay of the airflow velocity along both the *z*-axis (spinning line direction) and the *y*-axis (spinning line vertical direction). The airflow velocity decreases more rapidly in the *y*-axis. At *y* = 0.75 cm, the airflow velocity decreases from the maximum to 30 m/s, after which there is no longer significant decay. The airflow velocity in the *y*-axis direction causes the lateral movement of fibers and a smaller velocity decay leads the lateral movement of fibers to slow down. When the velocity in the *y*-axis direction is small, the lateral movement of fibers becomes slow. Under the influence of *z*-axis airflow velocity, the fibers move downward. In the melt-blowing airflow field, the airflow mainly moves in the *z*-axis direction and the airflow velocity decays more slowly in the *z*-axis direction. At *z* = 5 cm, the velocity decreases from the maximum to 30 m/s. This distance is very important for fiber drawing and attenuation. The high velocity of the airflow that can retain a larger distance is beneficial to fiber drawing and refinement.

**Figure 9:**
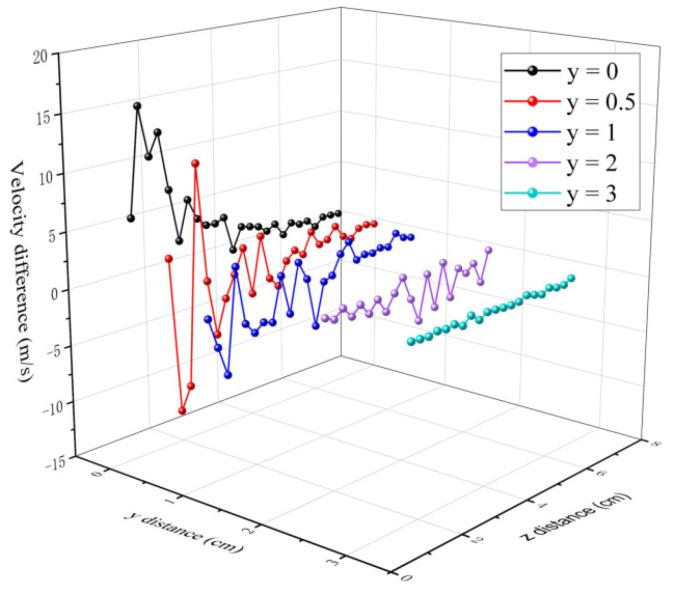
The variation of the velocity difference for the *y* line.

Figure 9 shows the variation of the velocity difference between each measurement point at the *y*-axis position, 0.1 cm, 0.5 cm, 1 cm, 2 cm, and 3 cm, respectively. The equation for the air velocity difference is expressed as

(1) $$∆V=V_{i}-V_{i-1}$$

where *i* is the measurement point on the *z*-axis from 0.75 cm to 7 cm.

At *y* = 0 cm, the air velocity on the centerline is decreasing along the *z*-axis. The air velocity difference ($∆V_{y=0}$ > 0) is a positive value and the fiber velocity increases as the air velocity decreases. At *y* = 0.5 cm and 1 cm, the $∆V_{y=0.5}$ and $∆V_{y=1}$ have positive and negative values, indicating that the velocity has acceleration and deceleration at a different *z* position. The air velocity difference can affect the fiber drawing ratio and cause the fiber to fluctuate. The movement trajectory direction of the fiber is changed with changes in the air velocity. At *z* < 5 cm, Figure 9 shows that there are large fluctuations in the airflow velocity difference at 0 < *y* < 1 cm. At *z* > 5 cm, the velocity difference becomes smaller. This is consistent with Zeng’s research which found that fibers quickly accelerate at about 5 cm distance from the die [42].

### 3.3. Fiber Motion in the Melt-Blowing Airflow Field

According to the kinetic theory of Entov [43] on the motion of the liquid jet in the airflow field, it is known that when the air velocity (U0) exceeds a critical value (U*), a small bending disturbance in the liquid jet appears and grows, gradually forming a bending instability motion. The critical value corresponds to the velocity at the beginning of the bending instability for the liquid jet, which can be calculated according to the following equation.

(2) $$U^{*}=\sqrt{\alpha /(\rho_{a}a_{0})}$$

where α is the liquid surface tension coefficient, $a_{0}$ is the radius of the undisturbed liquid jet, which is the inner radius of the nozzle in the experiment. $\rho_{a}$ is the density of the airflow. In this experiment, α = 0.70 kg/s^2^, $\rho_{a}$ = 1.29 kg/m^3^, and $a_{0}$ = 0.21 mm are calculated according to Equation (2) to obtain the critical value U* = 51 m/s [42].

Comparing the calculated critical velocity with the experimentally measured airflow velocity, the measured result shows that the airflow velocity along the *z*-axis is much higher than this critical value except for a very small distance from the die (*x* < 2 mm). It indicates that the bending unstable motion of the polymer jet occurs within a short time after it is extruded from die spinneret.

**Figure 10:**
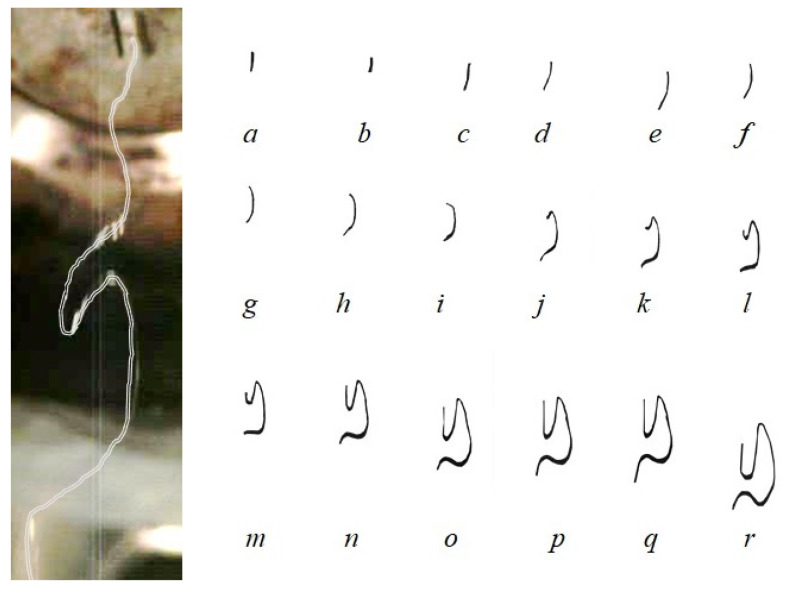
The formation of fiber loops in the melt-blowing airflow field. (**a**–**r**) represent the different morphologies of the fiber segments during the movement of the airflow field.

Figure 10 shows the trajectory of fiber in the melt-blowing airflow field. The formation of the fiber loop process and the movement of the fiber loop were expressed in the melt-blowing airflow field. In the vicinity of the spinneret, the fibers move vertically downward as straight segments, from Figure 10a–d. As seen in Figure 10e–i, the fiber shows a noticeable bend and its movement undergoes a gradual change with the spinning distance increasing. In the melt-blowing airflow field, the airflow velocity is high near the spinneret speed while the fiber velocity is low near the spinneret. There is a large speed difference between the fiber and the airflow. The fiber has a large acceleration, and the velocity of fiber movement increases more quickly. Thus, the motion below the fiber section lags behind the motion above the fiber section, causing the fiber to bend and create a semi-circular fiber loop. The fiber loop is in a high-velocity airflow field, and the perimeters of the loop increase as it is drawn and refined. The fiber diameter decreases and the fibers draw finer at the same time, as seen in Figure 10j–r. It is important to note that the shape of the fiber loops changes during the formation of the fiber loops. The shape change in the fiber loop has a large effect on the fiber motion and attenuation.

**Figure 11:**
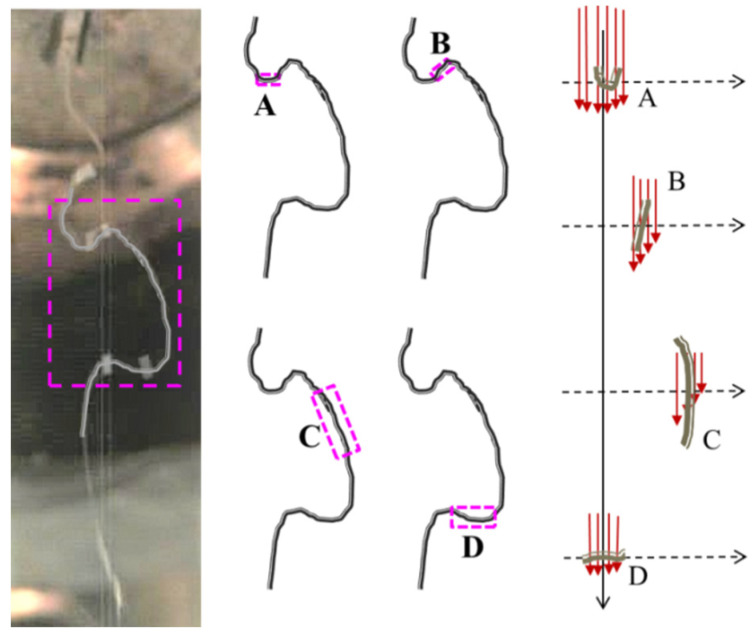
The relationship between the morphological structure of the fiber loop and the airflow in the melt-blowing process. (**A**) the initial section of the fiber loop, (**B**) the upper part of the fiber loop, (**C**) the part of the fiber loop parallel to the airflow velocity, (**D**) the end section of the fiber loop.

Figure 11 shows the melt-blowing fiber motion state in the airflow field. It can be seen that the fiber loop exists in different shapes at different positions. The purple box in the figure is a fiber loop, and is composed of the A, B, C, D fiber segments. The arrows in the Figure 11 represent the magnitude of the airflow velocity in the direction of the spinning line. The fiber segment A is in the initial section of the fiber loop perpendicular to the airflow velocity. The fiber segment B is the upper part of the fiber loop inclined to the airflow velocity. The fiber segment B and the airflow field between the existences of a certain angle. The fiber segment C is part of the fiber loop parallel to the airflow velocity, which is located far from the centerline. The fiber segment D is in the end section of the fiber loop and is also perpendicular to the airflow velocity. It should be noted that the velocity of the airflow differs depending on the position of fiber segment A, segment B, segment C, and segment D. The velocity for fiber segment A is the largest and there is a large downward motion velocity in this fiber section. The air velocity can be divided into longitudinal velocity and transverse velocity at fiber segment B. The longitudinal velocity has a stretching effect on the fiber. The transverse velocity moves the fiber away from the centerline position, thus making the fiber loop wider laterally. The fiber section C is located away from the centerline, where the airflow velocity is low. In this position, the fiber direction is consistent with the direction of airflow velocity and the fiber will also be drawn by the airflow and become thinner. Compared to the fiber section A, the airflow velocity at the position of the fiber section D is small. Although the fiber segment D is perpendicular to the airflow velocity and moves downward, it moves downward more slowly than the fiber segment A. As the distance increases, Figure 11 shows that the distance between the fiber segment A and the fiber segment D gradually decreases and also overtakes or crosses together because the fiber segment A moves faster than the fiber segment D.

**Figure 12:**
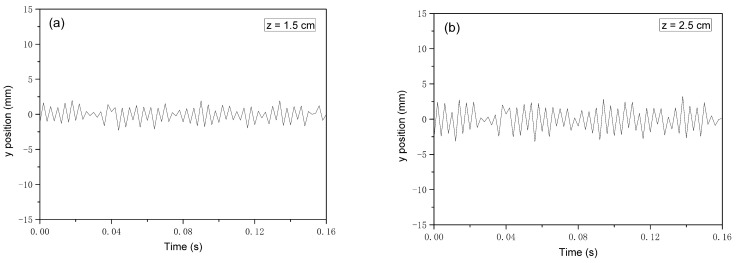

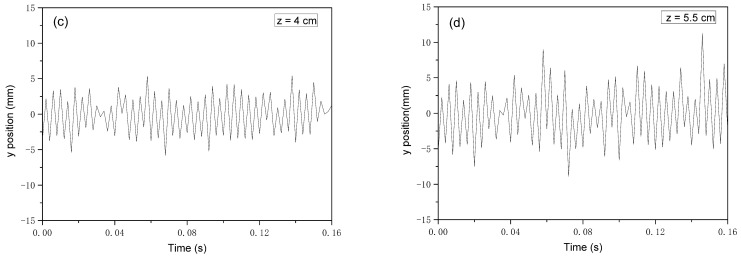
The fiber transverse displacement at different *z* positions in the full recording time of 0.16 s. (**a**) *z* = 1.5 cm, (**b**) *z* = 2.5 cm, (**c**) *z* = 4 cm, (**d**) *z* = 4 cm.

Figure 12 shows each data point corresponds to a fiber position determined from a frame captured by high-speed camera. The data were gathered by following motion from *−y* to *+y* at a constant *z* position over the full recording time of 0.16 s. It can be seen that there is a transverse displacement of the polymer jet during the fiber drawing process. The transverse displacement of the polymer jet is relatively small, close to the die, and increases as the distance from the die increases. When *z* = 1.5 cm, 2.5 cm, 4 cm, and 5.5 cm, the average fiber fluctuation amplitudes are 1.05 mm, 1.71 mm, 2.83 mm, and 3.97 mm, respectively. By observing the variation of fiber transverse displacement over the distance of the spinning line, it is possible to understand the refinement process of the polymer jet in the airflow field. The transverse displacement of the fiber can prolong the motion trajectory of the fiber and increases the drawing time. The oscillation amplitude affects the structure and performance of the melt-blowing web structure. It is useful information for designing and determining the optimal distance between the die and collector.

**Figure 13:**
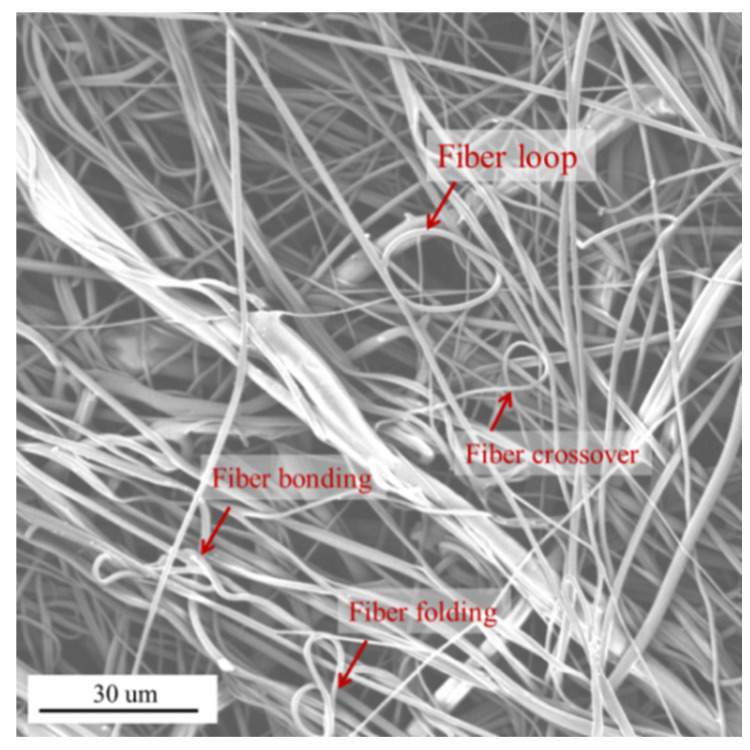
The morphology of fibers in the melt-blown web.

There are a number of reasons that can affect the trajectory of melt-blown fibers during forming and in defects in nonwovens, such as the polymer viscosity, intermolecular interaction of components, flow rate, and others. Some researchers [44,45,46] have noted that the melt-blowing airflow field determines the pattern of fiber motion behavior, which results in fibers and nonwoven morphologies. It should be noted that the melt-blowing fibers may cross and stack when moving in the airflow field, which can lead to changes in the magnitude and direction of the airflow velocity, and then the trajectory of the fibers can also change. When the fiber crossover point appears early on, the fibers will fuse together after crossing because the fibers still have a viscous flow. The result is that melting point or multiple merged fibers exist in melt-blown fabric. When the fiber crossover point appears late, the fibers are completely cooled. There are some bent and folded fibers in melt-blown fabric. These phenomena can be verified in the SEM of melt-blown web, as shown in Figure 13.

Figure 13 shows the morphology of fibers in the melt-blown web. It shows that the fibers in the web will have crossover, folding, and bonding points, which are caused by the different velocities between different fiber segments during the fiber movement. Figure 13 also shows that the fibers in the web are randomly distributed. During the melt-blowing fiber forming process, the velocity and direction of the airflow change with the direction of fiber movement, creating a turbulent airflow field. The turbulent airflow field will further contribute to a more random distribution of fibers. The interaction between airflow and fibers results in the melt-blowing fibers show a random arrangement in the web.

## 4. Conclusions

In summary, the three-dimensional airflow field of slot die (6.25 cm × 6 cm × 12 cm) is measured using a hot-wire anemometer experimental method. The airflow velocity is asymmetrically distributed in the melt blowing. As the die distance increases, the air velocity becomes smaller, and the air diffusion area becomes larger. The maximum airflow velocity exists near the die face, in the range of 130–160 m/s. In the region of −0.3 cm < *y* < 0.3 cm and 0 < *z* < 2 cm, the airflow has a high velocity (>100 m/s) and plays a major role in fiber drawing, forming, and movement. The fiber motion is captured by an online high-speed camera. It reveals that the airflow velocity, velocity difference, and dispersion area affect the fluctuations of fiber. At 1.5 cm, 2.5 cm, 4 cm, and 5.5 cm, the fiber fluctuation amplitude distributions are 1.05 mm, 1.71 mm, 2.83 mm, and 3.97 mm, respectively. The melt-blowing fibers move in a straight line in the initial section and then create fiber loops due to the airflow velocity distribution characteristics. The segments in the fiber loop have different morphologies and different positions in the airflow field, which can lead to different velocities of movement of the fibers, forming fiber crossings, folding, and bonding. The morphology of the fiber in the web verifies the fiber movement pattern in the melt-blowing airflow field. This work provides a better understanding of a melt-blowing airflow field and fiber formation.

## Figures and Tables

**Table 1:** The information of experimental studies on the melt-blowing airflow field.

Authors	Year	Measurement Instrument	Condition	Measurement Content
Uyttendaele and Shambaugh [36]	1989	Pitot tube	Isothermal	Velocity under an annular die
Majumda and Shambaugh [30]	1991	Pitot tube and thermocouple	Nonisothermal	Velocity and temperature under an annular die
Mohammed and Shambaugh [37]	1993	Pitot tube and thermocouple	Nonisothermal	Velocity and temperature under a multiorifice annular die
Harpham and Shambaugh [31]	1996	Pitot tube	Isothermal	Velocity under a slot die
Harpham and Shambaugh [32]	1997	Pitot tube and thermocouple	Nonisothermal	Velocity and temperature under a slot die
Tate and Shambaugh [38]	2004	Pitot tube and thermocouple	Nonisothermal	Effects of geometry parameters of slot dies on the air velocity and temperature
Moore [39]	2004	Pitot tube and thermocouple	Nonisothermal	Air velocity and temperature under a multiorifice slot die
Tan [40]	2012	Schlieren visualization	Nonisothermal	Air density oscillation
Xie [35]	2020	Particle Image Velocimetry	Nonisothermal	The turbulent Airflow in Slot die

**Table 2:** Dimensions (mm) of the slot die.

The Nose-Piece Width (*f*)	The Slot Angle (α)	The Slot Width (*e*)	The Slot Length (*l*)	The Orifice Diameter (*d*)
1.28	30°	0.65	6	0.42

## Data Availability

Data are contained within the article.
